# A Global Meta-Analysis on the Impact of Management Practices on Net Global Warming Potential and Greenhouse Gas Intensity from Cropland Soils

**DOI:** 10.1371/journal.pone.0148527

**Published:** 2016-02-22

**Authors:** Upendra M. Sainju

**Affiliations:** USDA, Agricultural Research Service, Northern Plain Agricultural Research Laboratory, 1500 North Central Avenue, Sidney, MT 59270, United States of America; North Carolina State University, UNITED STATES

## Abstract

Management practices, such as tillage, crop rotation, and N fertilization, may affect net global warming potential (GWP) and greenhouse gas intensity (GHGI), but their global impact on cropland soils under different soil and climatic conditions need further evaluation. Available global data from 57 experiments and 225 treatments were evaluated for individual and combined effects of tillage, cropping systems, and N fertilization rates on GWP and GHGI which accounted for CO_2_ equivalents from N_2_O and CH_4_ emissions with or without equivalents from soil C sequestration rate (ΔSOC), farm operations, and N fertilization. The GWP and GHGI were 66 to 71% lower with no-till than conventional till and 168 to 215% lower with perennial than annual cropping systems, but 41 to 46% greater with crop rotation than monocroppping. With no-till vs. conventional till, GWP and GHGI were 2.6- to 7.4-fold lower when partial than full accounting of all sources and sinks of greenhouse gases (GHGs) were considered. With 100 kg N ha^-1^, GWP and GHGI were 3.2 to 11.4 times greater with partial than full accounting. Both GWP and GHGI increased curvilinearly with increased N fertilization rate. Net GWP and GHGI were 70 to 87% lower in the improved combined management that included no-till, crop rotation/perennial crop, and reduced N rate than the traditional combined management that included conventional till, monocopping/annual crop, and recommended N rate. An alternative soil respiration method, which replaces ΔSOC by soil respiration and crop residue returned to soil in the previous year, similarly reduced GWP and GHGI by 133 to 158% in the improved vs. the traditional combined management. Changes in GWP and GHGI due to improved vs. traditional management varied with the duration of the experiment and inclusion of soil and climatic factors in multiple linear regressions improved their relationships. Improved management practices reduced GWP and GHGI compared with traditional management practices and combined management practices were even more effective than individual management practices in reducing net GHG emissions from cropland soils. Partial accounting overestimated GWP and GHGI values as sinks or sources of net GHGs compared with full accounting when evaluating the effect of management practices.

## Introduction

Agricultural management practices contribute from 6% of the total greenhouse gas (GHGs: CO_2_, N_2_O, and CH_4_) emissions in the USA [1] to about 20% globally [2]. The impact of these GHGs in radiative forcing in earth’s atmosphere is quantitatively estimated by calculating net global warming potential (GWP) which accounts for all sources and sinks of CO_2_ equivalents from farm operations, chemical inputs, soil C sequestration, and N_2_O and CH_4_ emissions [3, 4]. Another measure of GHGs’ impact is net greenhouse gas intensity (GHGI) which is expressed as net GWP per unit crop yield [4]. While soil C sequestration is the major sink and GHG emissions are sources of CO_2_ in agroecosystems that are affected by soil and climatic conditions and management practices [4, 5], machinery and inputs used for growing crops, such as tillage, planting, harvesting, and applications of fertilizers, herbicides, and pesticides, can produce CO_2_, thereby reducing the GHG mitigation potential [3, 6, 7]. The GWP and GHGI are typically controlled by the balance between soil C sequestration rate (ΔSOC), N_2_O and CH_4_ emissions, and crop yields [3, 4, 8].

Novel management practices that can mitigate GHG emissions and therefore GWP and GHGI include no-till, increased cropping intensity, diversified crop rotation, cover cropping, and reduced N fertilization rates [3, 4, 8]. No-till can increase soil organic C (SOC) compared with conventional till by reducing soil disturbance, residue incorporation, and microbial activity that lower CO_2_ emissions [4, 9]. Diversified cropping systems, such as intensive cropping, crop rotation, and cover cropping, can increase SOC by increasing the quality and quantity of crop residue returned to the soil compared with less diversified systems, such as crop-fallow, monocropping, and no cover crop [4, 10]. Nitrogen fertilization typically stimulates N_2_O emissions [3, 11], but can have a variable effect on CO_2_ and CH_4_ emissions [10, 12]. Because N_2_O emissions plays a major role in enhancing GWP and GHGI, practices that can reduce N fertilization rates without influencing crop yields can substantially reduce net GHG emissions [3, 4].

Management practices used for GHG mitigation sometime can have counter effects. For example, no-till can increase N_2_O emissions compared with conventional till in humid regions by increasing soil water content and denitrification, thereby offsetting the GHG mitigation potential [13]. Incorporation of root residue can increase soil C sequestration, but root respiration and mineralization of crop residue and SOC can have negative impacts on GHG mitigation [10, 14]. Besides the direct effect of N fertilization on N_2_O emissions, indirect effects, such as NH_4_ volatilization, N leaching, and urea hydrolysis in the soil can also counteract the mitigation potential [15]. All of these factors should be considered while calculating net GWP and GHGI, regardless of management practices [3, 4, 16].

Several methods have been employed to calculate GWP and GHGI by using the SOC method which considers ΔSOC as CO_2_ sink. Some have used the sum of CO_2_ equivalents of N_2_O and CH_4_ emissions [17, 18, 19], while others [20, 21] have included CO_2_ equivalents of all three GHGs. Still others have used CO_2_ equivalents of N_2_O and CH_4_ emissions and ΔSOC [22, 23, 24]. A full accounting of all sources and sinks of GHGs to calculate net GWP and GHGI includes CO_2_ equivalents from farm operations, N fertilization, and other inputs in addition to above parameters [3, 4, 8, 16, 25, 26, 27, 28, 29, 30]. Some have excluded N_2_O and CH_4_ emissions, but used CO_2_ equivalents of all other sources and sinks [7]. An alternative method (hereby called the soil respiration method) of calculating GWP and GHGI includes substituting ΔSOC by soil respiration and the amount of previous year’s crop residue returned to the soil [4, 27, 28, 29, 30, 31]. Each method has its own advantages and drawbacks which will be explored in detail by comparing GWP and GHGI values below.

Information on the effects of soil and crop management practices on GHG emissions in croplands is available [17, 32, 33, 34]. Relatively, little is known about the influence of management practices on GWP and GHGI. The objectives of this study were to: (1) conduct a meta-analysis of global data available in the literature on the individual and combined effects of tillage, cropping systems, and N fertilization rates on GWP and GHGI calculated by the SOC method in cropland soils using partial or full accounting of all sources and sinks of GHGs, (2) relate GWP and GHGI with the duration of the experiment and soil and climatic conditions, (3) compare GWP and GHGI values calculated by the SOC and soil respiration methods, and (4) identify improved management practices that can reduce net GWP and GHGI.

## Materials and Methods

### Data collection and management

**Fig 1:**
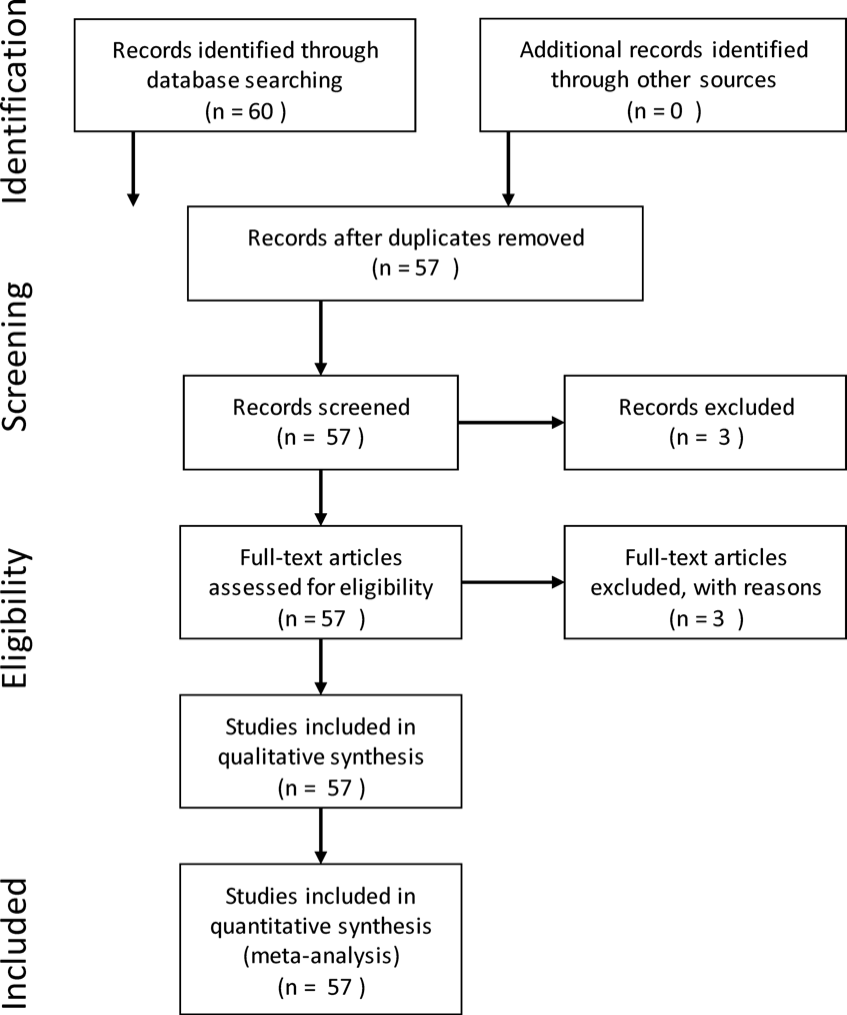
The PRISMA (Preferred Reporting Items for System Review and Meta-Analysis) guidelines used collection and meta-analysis of data.

Data on GWP and GHGI were pulled from the available literature from 57 experiments and 225 treatments from national and international regions and grouped by individual and combined management practices that included tillage, cropping systems, and N fertilization rates (Tables 1, 2, 3, 4 and 5) using all available resources (e.g. Web of Science, Goggle Scholar, SCOPUS, etc.). The PRISMA (Preferred Reporting Items for System Review and Meta-Analysis) guidelines (Fig 1) have been followed for collection and meta-analysis of data. In each management group, GWP and GHGI values were listed based on soil and climatic conditions, cropping systems, duration of study in each location, and parameters used for calculations. In some studies where two or more treatments were arranged in split-plot arrangements to evaluate the effects of individual and combined management practices on GWP and GHGI, values for main and split-plot treatments were used as individual management practices and their interactions as combined management practices when data are significantly different among treatments and interactions using General Linear Model (GLM) and mean separation tests. For experiments with unbalanced treatments, main treatment was considered as individual management practice when data were analyzed using the orthogonal contrast test and other mixed treatments as combined management practices when analyzed using GLM and mean separation tests, provided that differences among treatments are significant. When main treatments cannot be separated in a combination of various treatments, such treatments were considered as combined management practices and data were analyzed as above.

**Table 1:** Effect of tillage on net global warming potential (GWP) and greenhouse gas intensity (GHGI) calculated by using the soil organic C method in various regions with different soil and climatic conditions.

Location	Soil type	Annual precip.	Study duration	Mean air temp.	Tillage†	GWP‡	GHGI‡	Parameters used to calculate GWP/GHGI	Reference
		mm	yr	°C		kg CO_2_ eq. ha^-1^ yr^-1^	kg CO_2_ eq. Mg^-1^ grain or biomass		
Colorado, USA	Clay loam	382	5	10.6	NT	-15	18	N_2_O, CH_4_, ΔSOC, farm operation, inputs	[26]
					CT	1479	143		
Colorado, USA	Clay loam	382	3	10.6	NT	-516	-60	N_2_O, CH_4_, ΔSOC, farm operation, inputs	[4]
					CT	1071	93		
Queensland, Australia	Clay	728	4	17.2	NT	403	158	N_2_O, CH_4_, ΔSOC	[23]
					CT	495	195		
Michigan, USA	Sandy loam, loam	890	1	9.7	NT	2870	----	CO_2_, CH_4_, N_2_O	[21]
					CT	11500	----		
Parana, Brazil	Clay	1400	1	23.0	NT	-500	-32	N_2_O, CH_4_, ΔSOC	[24]
					CT	2900	172		
Michigan, USA	Sandy loam, loam	890	9	9.7	NT	140	----	N_2_O, CH_4_, ΔSOC, farm operation, inputs	[16]
					CT	1140	----		
Hyderabad, India	Clay	1520	20	25.0	NT	8930	----	N_2_O, CH_4_, ΔSOC, farm operation, inputs	[16]
					CT	10250	----		
Colorado, USA	Clay loam	890	1	10.6	NT	-1253	----	N_2_O, CH_4_, ΔSOC, farm operation, inputs, irrigation	[8]
					CT	2264	----		
North Dakota, USA	Sandy loam	373	4	5.2	NT	887	420	N_2_O, CH_4_, ΔSOC, farm operation, inputs, irrigation	[29]
					CT	1287	655		

†Tillage are CT = conventional till, NT = no-till.

‡Positive values indicate source and negative values sink of CO_2_.

**Table 2:** Effect of cropping systems on net global warming potential (GWP) and greenhouse gas intensity (GHGI) calculated by using the soil organic C method in various regions with different soil and climatic conditions.

Location	Soil type	Annual precip.	Mean air temp	Study duration	Cropping system†	GWP‡	GHGI‡	Parameters used to calculate GWP/GHGI	Reference number
		mm	°C	yr		kg CO_2_ eq. ha^-1^ yr^-1^	kg CO_2_ eq. Mg^-1^ grain or biomass		
Colorado, USA	Clay loam	382	10.6	5	C	222	65	N_2_O, CH_4_, ΔSOC, farm operation, inputs	[26]
					C-S	508	60		
Colorado, USA	Clay loam	382	10.6	3	C	-557	-64	N_2_O, CH_4_, ΔSOC, farm operation, inputs	[4]
					C-S	104	42		
Michigan, USA	Sandy loam, loam	890	9.7	1	G	-3500	----	CO_2_, CH_4_, N_2_O,	[21]
					A	-20	----		
					PO	-105	----		
Saskatche-wan, Canada	Loam, silt loam	250	5.2	3	W-W	578	----	N_2_O, ΔSOC, farm operation	[47]
					W-L	396	----		
					W-Fx-W-P	779	----		
					W-Fx-W-W	953	----		
Nebraska, USA	Silty clay loam	600	11.0	1.5	C	690	48	N_2_O, CH_4_, ΔSOC, farm operation, inputs, irrigation	[25]
					C-S	1020	102		
Michigan, USA	Sandy loam	890	9.7	1	C-W-S	640	----	N_2_O, CH_4_, ΔSOC, farm operation, inputs, irrigation	[8]
					Af	-200	----		
Colorado, USA	Clay loam	382	10.6	1	W-C-F	254	----	N_2_O, CH_4_, ΔSOC, farm operation, inputs,	[8]
					C	-498	----		
					G	-642	----		
Colorado, USA	Clay loam	382	10.6	1	C	-1291	----	N_2_O, CH_4_, ΔSOC, farm operation, inputs, irrigation	[8]
					C-S	-553	----		
Central North Dakota, USA	Silt loam	407	6.0	1.5	W-F	1654		N_2_O, CH_4_, and ΔSOC	[52]
					W-SF-RY	1660			
Western North Dakota, USA	Sandy loam	373	5.2	4	B	971	300	N_2_O, CH_4_, ΔSOC, farm operation, inputs, irrigation	[29]
		373			B-P	771	250		
Western Montana, USA	Silt loam	453	6.2	2	Af	2187	310	N_2_O, CH_4_, ΔSOC, farm operation, inputs	[28]
					W	5074	730		
					W-P/B-F	5191	1065		

† Crops are A = alfalfa, B = barley, C = corn, F = fallow, Fx = flax, G = grass, P = pea, PO = poplar, L = lentil, S = soybean. Letters joined by hyphenation indicates crop rotation, e.g. W-F = wheat-fallow rotation.

‡ Positive values indicate source and negative values sink of CO_2_.

**Table 3:** Effect of N fertilization rate on net global warming potential (GWP) and greenhouse gas intensity (GHGI) calculated by using the soil organic C method in various regions with different soil and climatic conditions.

Location	Soil type	Annual precip.	Mean air temp.	Study duration	Crop	N rate	GWP†	GHGI†	Parameters used to calculate GWP/GHGI	Reference
		mm	°C	yr		kg N ha^-1^	kg CO_2_ eq. ha^-1^ yr^-1^	kg CO_2_ eq. Mg^-1^ grain or biomass		
Colorado, USA	Clay loam	382	10.6	5	Corn, soybean	0	472	77	N_2_O, CH_4_, ΔSOC, farm operation, inputs	[26]
						134	542	45		
						246	1197	102		
Colorado, USA	Clay loam	382	10.6	3	Corn, soybean	0	-77	-19	N_2_O, CH4, ΔSOC, farm operation, inputs	[4]
						134	449	-32		
						246	500	37		
Queensland, Australia	Clay	728	17.2	4	Wheat	0	324	139	N_2_O, CH_4_, ΔSOC	[23]
						90	575	214		
California, USA	Clay	368	15.0	2	Rice	0	3965	861	N_2_O, CH_4_	[19]
						80	4789	544		
						140	5437	463		
						200	5395	410		
						260	5507	445		
Colorado, USA	Clay loam	382	10.6	1	Corn, soybean	0	-311	----	N_2_O, CH_4_, ΔSOC, farm operation, inputs, irrigation	[8]
						134	629	----		
						202	595	----		
California,	Clay	368	15.0	3	Rice	0	658	156	N_2_O, CH_4_	[18]
USA						50	816	120		
						100	712	91		
						150	1491	188		
						200	1541	190		
California, USA	Clay loam	368	15.0	3	Rice	0	5061	844	N_2_O, CH_4_	[18]
						50	6012	772		
						100	6768	687		
Arkansas, USA	Silt loam	1200	12.5	3	Rice	0	1068	278	N_2_O, CH_4_	[18]
						112	2018	265		
						168	2069	257		
						224	2238	286		
North Dakota, USA	Sandy loam	373	5.2	4	Barley, pea	0	926	617	N_2_O, CH_4_, ΔSOC, farm operation, inputs, irrigation	[29]
						101	1248	383		
Montana, USA	Loam	350	6.2	4	Barley, pea	0	635	453	N_2_O, CH_4_, ΔSOC, farm operation, inputs	[30]
						80	185	105		
Beijing,China	Loam	600	10.0	6	Corn, wheat	0	2702	369	N_2_O, CH_4_, ΔSOC, farm operation, inputs, irrigation	[54]
						247	2853	255		
						280	4309	350		
Nanjing, China	Silt loam	1107	15.4	2	Amaranth, Tug choy, Bok choy, Corriander	0	-2347	-139	N_2_O, CH_4_, ΔSOC, farm operation, inputs	[55]
						1475	-1650	5		
						1967	7700	109		
Nanjing, China	Silt clay	1107	15.4	2	Rice, wheat	0	5700	740	N_2_O, CH_4_, ΔSOC	[56]
						360	7210	500		
						432	6660	410		
						480	8360	580		

† Positive values indicate source and negative values sink of CO_2_.

**Table 4:** Effect of combined management practices (tillage, cropping system, and N fertilization) on net global warming potential (GWP) and greenhouse gas intensity (GHGI) calculated by using the soil organic C method in various regions with different soil and climatic conditions.

Location	Soil type	Annual precip.	Mean air temp.	Study duration	Combined management practices	GWP†	GHGI†	Parameters used to calculate GWP/GHGI	Reference
		mm	°C	yr		kg CO_2_ eq. ha^-1^ yr^-1^	kg CO_2_ eq. Mg^-1^ grain or biomass		
Colorado, USA	Clay loam	382	10.6	5	CT-CC-N0‡	709	108	N_2_O, CH_4_, ΔSOC, farm operation, inputs	[26]
					CT-CC-N1‡	1545	136		
					CT-CC-N2‡	2184	185		
					NT-CC-N0‡	234	46		
					NT-CC-N1‡	-459	-47		
					NT-CC-N2‡	210	19		
					NT-CB-N0‡	33	6		
					NT-CB-N2‡	983	113		
Colorado, USA	Clay loam	382	10.6	3	CT-CC-N0‡	80	13	N_2_O, CH_4_, ΔSOC, farm operation, inputs	[4]
					CT-CC-N1‡	1333	117		
					CT-CC-N2‡	1800	150		
					NT-CC-N0‡	-233	-50		
					NT-CC-N1‡	-436	-53		
					NT-CC-N2‡	-880	-77		
					NT-CB-N0‡	139	127		
					NT-CB-N2‡	68	-43		
Queensland, Australia	Clay	728	17.2	4	CT-SB-N0§	277	117	N2O, CH4, ΔSOC	[23]
					CT-SB-N90§	710	272		
					CT-SR-N0§	338	148		
					CT-SR-N90§	654	243		
					NT-SB-N0§	329	136		
					NT-SB-N90§	534	202		
					NT-SR-N0§	350	153		
					NT-SR-N90§	401	140		
Nebraska, USA	Silty clay loam	600	11.0	1.5	CC-F1¶	540	39	N_2_O, CH_4_, ΔSOC, farm operation, inputs, irrigation	[25]
					CC-F2¶	840	56		
					CB-F1¶	1020	104		
					CB-F2¶	1020	99		
Colorado, USA	Clay loam	382	10.6	1	CT-CC-N0‡	1647	**----**	N_2_O, CH_4_, ΔSOC, farm operation, inputs, irrigation	[8]
					CT-CC-N1‡	2383	**----**		
					CT-CC-N2‡	2763	**----**		
					NT-CC-N0‡	-1766	**----**		
					NT-CC-N1‡	-1125	**----**		
					NT-CC-N2‡	-815	**----**		
					NT-CB-N0‡	-942	**----**		
					NT-CB-N2‡	-164	**----**		
Minnesota, USA	Loam, silty, clay loam, clay loam	645	4.3	3	BAU#	5000	1094	N_2_O, CH_4_, ΔSOC, farm operation, inputs	[27]
					MAXC#	3500	978		
					OGGB#	4000	1183		
North Dakota, USA	Sandy loam	373	5.2	4	IR-CT-B-NF††	1607	450	N_2_O, CH_4_, ΔSOC, farm operation, inputs, irrigation	[29]
					IR-CT-B-NO††	1099	730		
					IR-NT-BP-NF††	1045	290		
					IR-NT-B-NF††	1117	320		
					IR-NT-B-NO††	952	670		
					NIR-CT-B-NF††	1443	480		
					NIR-CT-B-NO††	998	660		
					NIR-NT-BP-NF††	496	210		
					NIR-NT-B-NF††	824	280		
					NIR-NT-B-NO††	656	410		
Eastern Montana, USA	Loam	350	6.2	4	CT-BF-N0‡‡	1153	836	N_2_O, CH_4_, ΔSOC, farm operation, inputs	[30]
					CT-BF-N80‡‡	403	280		
					NT-BP-/N0‡‡	120	86		
					NT-BP-/N80‡‡	110	58		
					NT-B-N0‡‡	632	446		
					NT-B-N80‡‡	43	23		
Western Montana, USA	Silt loam	453	5.5	2	HA-A§§	927	150	N_2_O, CH_4_, ΔSOC, farm operation, inputs	[28]
					HA-W§§	5500	730		
					HA-WP/BF§§	3638	650		
					SHG-A§§	3447	470		
					SHG-W§§	4647	430		
					SHG-WP/BF§§	7031	1480		

† Positive values indicate source and negative values sink of CO_2_.

‡ CT = conventional till, NT = no-till, CC = continuous corn, CB = corn-soybean rotation, N0 = 0 kg N ha^-1^, N1 = 134 kg N ha^-1^, and N2 = 56–246 kg N ha^-1^.

§ CT = conventional till, NT = no-till, SB = stubble burned, SR = stubble retained in the soil, N0 = 0 kg N ha^-1^, N90 = 90 kg N ha^-1^.

¶ CC = continuous corn, CB = corn-soybean, F1 = 130–140 kg N ha^-1^, 0 kg P ha^-1^, 0 kg K ha^-1^; F2 = 230–310 kg N ha^-1^, 45 kg P ha^-1^, 85 kg K ha^-1^.

# BAU = conventional till corn-soybean rotation with 143 kg N ha^-1^, 17 kg P ha^-1^, and 0 kg K ha^-1^; MAXC = strip till corn-soybean-wheat/alfalfa-alfalfa rotation with 89 kg N ha^-1^, 32 kg P ha^-1^, and 28 kg K ha^-1^; OGCB = strip till corn-soybean-wheat/alfalfa-alfalfa rotation with 0 kg N ha^-1^, 0 kg P ha^-1^; and 0 kg K ha^-1^.

†† IR = irrigated, NIR = nonirrigated, CT = conventional till, NT = no-till, B = malt barley, BP = malt barley-pea rotation, NO = 0 kg K ha^-1^, and NF = 67–134 kg K ha^-1^.

‡‡ CT = conventional till, NT = no-till, B = malt barley, BF = malt barley-fallow rotation, BP = malt barley-pea rotation, N0 = 0 kg K ha^-1^, N80 = 0 kg K ha^-1^.

§§ HA = herbicide application for weed control, SHG = sheep grazing for weed control, A = alfalfa, W = wheat, WP/BF = wheat-pea/barley mixture hat-fallow rotation.

**Table 5:** Soil respiration method of calculating net global warming potential (GWP) and greenhouse gas intensity (GHGI) in various regions with different soil and climatic conditions as affected by combined management practices (tillage, cropping system, and N fertilization).

Location	Soil type	Annual precip.	Mean air temp.	Study duration	Combined management practices	GWP†	GHGI†	Parameters used to calculate GWP/GHGI	Reference
		mm	°C	yr		kg CO_2_ eq. ha^-1^ yr^-1^	kg CO_2_ eq. Mg^-1^ grain or biomass		
Colorado, USA	Clay loam	382	10.6	3	CT-CC-N0‡	1953	133	Soil respiration, N_2_O, CH_4_, crop residue, farm operation, inputs	[4]
					CT-CC-N1‡	-1367	-45		
					CT-CC-N2‡	-1743	-162		
					NT-CC-N0‡	-833	-217		
					NT-CC-N1‡	-2990	-310		
					NT-CC-N2‡	-4300	-390		
					NT-CB-N0‡	9495	1340		
					NT-CN-N2‡	9850	865		
Arkansas, USA	Silt loam	1200	14.5	4	NIR-C§	-1351	----	N_2_O, CH_4_, crop residue, farm operation, inputs, irrigation	[31]
					NIR-CT§	760	----		
					IR-CT§	951	----		
					NIR-SO	-455	----		
					IR-SO§	-965	----		
					NIR-S§	-301	----		
					IR-S§	-4	----		
					IR-R§	6632	----		
					NIR-W§	661	----		
Minnesota, USA	Loam, silt, clay loam, clay loam	645	4.3	3	BAU¶	500	109	N_2_O, CH_4_, crop residue, farm operation, inputs	[27]
					MAXC¶	9100	2542		
					OGGB¶	1220	361		
North Dakota, USA	Sandy loam	373	5.2	4	IR-CT-B-NF#	-7793	-1950	Soil respiration N_2_O, CH_4_, crop residue, farm operation, inputs, irrigation	[29]
					IR-CT-B-NO#	-1495	-490		
					IR-NT-BP-NF#	-9169	-2490		
					IR-NT-B-NF#	-8112	-2150		
					IR-NT-B-NO#	-117	-10		
					NIR-CT-B-NF#	-7050	-2270		
					NIR-CT-B-NO#	-1752	-670		
					NIR-NT-BP-NF#	-6618	-2920		
					NIR-NT-B-NF#	-6243	-1920		
					NIR-NT-B-NO#	-1281	-510		
Eastern Montana, USA	Loam	350	6.2	4	CT-BF-N0††	114	83	Soil respiration, N_2_O, CH_4_, crop residue, farm operation, inputs	[30]
					CT-BF-N80††	-292	-203		
					NT-BP-/N0††	-1902	-1156		
					NT-BP-/N80††	-2107	-1109		
					NT-B-N0††	-574	-404		
					NT-B-N80††	-1944	-1002		
Western Montana, USA	Silt loam	453	5.5	2	HA-A‡‡	4970	780	Soil respiration, N_2_O, CH_4_, crop residue, farm operation, inputs	[28]
					HA-W‡‡	8740	1180		
					HA-WP/BF‡‡	5894	1040		
					SHG-A‡‡	7030	650		
					SHG-W‡‡	8574	1440		
					SHG-WP/BF‡‡	8868	1860		

† Positive values indicate source and negative values sink of CO_2_.

‡ CT = conventional till, NT = no-till, CC = continuous corn, CB = corn-soybean rotation, N0 = 0 kg N ha^-1^, N1 = 134 kg N ha^-1^, and N2 = 56–246 kg N ha^-1^.

§ IR = irrigated, NIR = nonirrigated, C = corn, CT = cotton, SO = sorghum, S = soybean, R = rice, and W = wheat.

¶ BAU = conventional till corn-soybean rotation with 143 kg N ha^-1^, 17 kg P ha^-1^, and 0 kg K ha^-1^; MAXC = strip till corn-soybean-wheat/alfalfa-alfalfa rotation with 89 kg N ha^-1^, 32 kg P ha^-1^, and 28 kg K ha^-1^; OGCB = strip till corn-soybean-wheat/alfalfa-alfalfa rotation with 0 kg N ha^-1^, 0 kg P ha^-1^; and 0 kg K ha^-1^.

# IR = irrigated, NIR = nonirrigated, CT = conventional till, NT = no-till, B = malt barley, BP = malt barley-pea rotation, NO = 0 kg K ha^-1^, and NF = 67–134 kg K ha^-1^.

†† CT = conventional till, NT = no-till, B = malt barley, BF = malt barley-fallow rotation, BP = malt barley-pea rotation, N0 = 0 kg K ha^-1^, N80 = 0 kg K ha^-1^.

‡‡ HA = herbicide application for weed control, SHG = sheep grazing for weed control, A = alfalfa, W = wheat, WP/BF = wheat-pea/barley mixture hat-fallow rotation.

The GWP using the SOC method [3, 4, 29, 30] to compare the effect of management practices in data analysis included the following options for calculations:

Partial accounting data:

Partialaccountingdata:GWP=CO2equivalentsfrom(N2O+CH4)emissionswithorwithoutCO2equivalentfromΔSOC. (1)

Fullaccountingdata:GWP=CO2equivalentsfrom(N2O+CH4)emissions+CO2equivalentsfrom(farmoperations+Nfertilization+otherinputs)−CO2equivalentfromΔSOC (2)

All data: GWP = CO_2_ equivalents calculated both from partial and full accounting data. The purpose of using all data option in the analysis was to compare them with partial and full accounting data options and to examine if the relationships of GWP and GHGI with management practices can be improved when the all data option was used compared with using only partial and full accounting data options.

In the soil respiration method [4, 29, 30, 31], GWP was calculated as:

$$GWP=CO_{2}\;equivalents\;from\;\left(CO_{2}\;\left[excluding\;root\;respiration\right]\;+\;N_{2}O\;+\;CH_{4}\right)\;emissions\;+CO_{2}\;equivalents\;from\;\left(farm\;operations\;+\;N\;fertilization\;+\;other\;inputs\right)\;-\;CO_{2}\;equivalent\;from\;previous\;year’s\;crop\;residue\;returned\;to\;the\;soil$$ (3)

The GHGI in the SOC or the soil respiration method was calculated as:

GHGI=GWP(SOCorsoilrespirationmethod)/grainorbiomassyield. (4)

Although data from 60 experiments and 255 treatments were collected, only data from 57 experiments and 225 treatments were selected for meta-analysis which meets the specific criteria shown below:

1. All experiments should be conducted in croplands in the field. Croplands included both uplands and lowlands under all agricultural irrigated and dryland crops. Number of crops grown in a year should be two or less. Data on GWP and GHGI estimated by models were excluded for analysis.
2. Treatments in the experiments should be replicated, randomized, and arranged in a proper experimental design.
3. For GWP and GHGI calculated by using the SOC method, soil C sequestration should have occur to a depth of 20 cm from the initiation of the experiment to the end of GHG measurement period. In the soil respiration method, experiments should have started in the previous year where the amount of crop residue returned to the soil was known.
4. Measurement of GHG emissions should occur at regular events, including close measurements during episodic events, such as immediately following precipitation, irrigation, tillage, fertilization, and snow melts.
5. A time horizon of 100 yr should be used to calculate the CO_2_ equivalents of N_2_O and CH_4_ emissions which have 298 and 25 times, respectively, more global warming potential than CO_2_ [35].
6. Carbon dioxide emissions associated with use of farm equipment for irrigation, tillage, fertilization, planting, herbicide and pesticide application, and harvest as well as manufacture and application of fertilizers and other chemical inputs should be either calculated based on the number of hours the equipment were used multiplied by CO_2_ emissions per liter of fuel or estimated as shown by various researchers [7, 36, 37].
7. For comparing the effects of individual and combined management practices on GWP and GHGI, the SOC method was used to calculate these parameters. Because of the limited availability of data, the soil respiration method was used only to evaluate the effect of combined management practice on GWP and GHGI.

### Statistical analysis of data

Meta-analysis of data was conducted by using procedure as suggested by various researchers [38, 39, 40, 41]. Those data where excessive levels of GHGs were reported, such as due to high rates of N fertilization, were discarded for analysis. A paired t-test was used for data analysis to compare the effects of individual and combined treatments of improved vs. traditional management practices on GWP and GHGI by evaluating significant difference between practices [42]. Individual improved management practices included no-till, crop rotation, increased cropping intensity, perennial crops, and reduced N fertilization rates. Individual traditional management practices included conventional till, monocropping, reduced cropping intensity, annual crops, and recommended N fertilization rates. Combined improved or traditional management practices included combinations of two or more of these practices. Comparisons included no-till vs. conventional till, crop rotation vs. monocropping, annual vs. perennial crop, and combined improved vs. combined traditional management practice that included a combination of these practices with or without cropping intensity and N fertilization rates. For cropping intensity and N fertilization rates, regression analysis were conducted to determine their relationships with GWP and GHGI [42]. For comparison of combined improved vs. combined traditional management practice, appropriate combination of individual treatments with lower and higher GWP and GHGI, respectively, were selected.

Regression analysis was used to relate changes in GWP and GHGI due to improved vs. conventional management with duration of the experiment to examine if the changes vary with time [42]. Multiple linear regressions were conducted to include soil and climatic factors (total annual precipitation and mean air temperature) in these analyses to determine if the relationships can be improved. For analysis of the soil factor, soil texture was assigned to a numerical value: coarse = 0, medium = 1, and fine = 2; where coarse refers to sand, loamy sand, and sandy loam; medium refers to loam, silt, silt loam, sandy clay, and sandy clay loam; and fine refers to clay, silty clay, clay loam, and silty clay loam [39].

## Results and Discussion

### Effect of tillage

A meta-analysis of nine experiments (Table 1) on the effect of tillage, when other practices, such as cropping systems, fertilization, and farm activities were similar between tillage systems, showed that no-till reduced GWP by 66% and GHGI by 71% compared with conventional till when the all data option of the SOC method of calculating GWP and GHGI was used (Table 6). Using the full accounting data option, no-till reduced GWP by 55% and GHGI by 58% compared with conventional till. With the partial accounting data option, the reductions in GWP and GHGI due to no-till vs. conventional till were 81 and 73%, respectively. Differences in crop yields among cropping systems and variations in soil and climatic conditions among regions resulted in different proportion of reductions in GWP and GHGI due to no-till vs. conventional till. Variability in GWP and GHGI were high, with coefficient of variation ranging from 19 to 91%.

**Table 6:** Effect of various management practices on net global warming potential (GWP) and greenhouse gas intensity (GHGI) based on the meta-analysis. Values for difference between practices are denoted as mean (± standard error).

Management practice	GWP		GHGI	
	N†	Difference between practices	N	Difference between practices
		kg CO_2_ eq. ha^-1^ yr^-1^		kg CO_2_ eq. Mg^-1^ grain or biomass
Tillage (NT vs. CT)‡				
All data	9	1212–3598 = -2386* (±874)	6	292–1008 = -716 (±566)
Full accounting data	6	1362–2915 = -1553* (±430)	3	126–297 = -171* (±33)
Partial accounting data	3	924–4965 = -4041 (±2485)	3	458–1719 = -1261 (±1142)
Cropping system§				
Crop rotation vs. monocrop	11	987–674 = 313** (±84)	11	304–215 = 89 (±34)
Corn-soybean vs. continuous corn	4	270 –(-234) = 504* (±114)	4	68–16 = 52 (±52)
Small grain-legume vs. continuous small grain	3	649–834 = -185** (±8)	3	----#
Cropping intensity (1.00 vs. 0.67)	11	827–1319 = -492 (±301)	6	225–572 = -347** (±64)
Cropping intensity (1.00 vs. 0.50)	11	827–853 = -26 (±426)	6	-----
Cropping intensity (0.67 vs. 0.50)	11	1319–853 = 466 (±21)	6	-----
Perennial vs. annual crop	11	(-604)– 885 = -1489*** (±278)	6	(-298)– 260 = -558** (±138)
Combined management practice¶				
Improved vs. traditional (SOC method)	9	297–2474 = -2177* (±671)	8	174–582 = -408* (±154)
Improved vs. traditional (Respiration method)	6	(-1909)– 5741 = -7650** (±1720)	5	-(620)– 1068 = -1688** (±315)

*Significant at *P* ≤ 0.05

**Significant at *P* ≤ 0.01

***Significant at *P* ≤ 0.001.

† Number of experiments included in the meta-analysis.

‡ Tillage is CT, conventional tillage; and NT, no-tillage. Full accounting data denotes calculation of GWP and GHGI by accounting all sources and sinks of GHGs (N_2_O and CH_4_ emissions, farm operations, inputs, and soil C sequestration). Partial accounting data denotes partial accounting of sources and sinks (N_2_O and CH_4_ emissions and/or soil C sequestration). All data denotes inclusions of both full and partial accounting.

§ Small grains include wheat and barley. Cropping intensity was calculated based on number of crops grown in a year.

¶ Combined management practices include combinations of tillage, cropping system, and N fertilization. Improved and traditional management practices were treatments with lowest and highest GWP and GHGI that were calculated by the soil organic C (SOC) and soil respiration method, respectively.

# Insufficient data.

It is not surprising to obtain high variability in GWP and GHGI due to management practices because of extreme variations in GHG values caused by large fluxes from episodic events of tillage, fertilization, precipitation, and snow melt, while variations from farm operations, N fertilization, and other inputs are low [3, 4, 8, 29, 30]. As a result, variability in GWP and GHGI calculated by the partial accounting data option (62 to 91%) was higher than calculated by the full (19 to 28%) accounting data option. The GHG emissions vary not only from one region to another due to differences in soil and climatic conditions, but also by diurnally, seasonally, and annually due to changes in soil temperature and water content in the same region [8, 16, 43]. Reductions in GWP and GHGI in no-till compared with conventional till, regardless of the option used for calculation, showed that increased C sequestration due to reduced soil disturbance and C mineralization reduced GWP and GHGI in no-till [3, 16, 44]. Conventional till increases crop residue incorporation and microbial activity, thereby reducing C sequestration, but increasing GWP and GHGI compared with no-till [8, 45].

Lower GWP and GHGI values due to no-till vs. conventional till in the partial than the full accounting data shows that the partial accounting data calculated greater GHG sink due to the effect of tillage than the full accounting data. This is because CO_2_ equivalents from farm operations, N fertilization, and other inputs were not accounted in the partial accounting data. Because all other farming operations were similar, the difference between no-till and conventional till was the use of several tillage operations to prepare a seed bed in conventional till, while soil was not disturbed in no-till. As a result, CO_2_ emissions associated with burning of fossil fuel for tractor operation was higher in conventional till than in no-till. Also, ΔSOC was lower in conventional till than no-till. The result was greater differences in GWP and GHGI values between no-till and conventional till in the partial than the full accounting data. It was not surprising that GWP and GHGI values due to no-till vs. conventional till calculated by the all data option were between full and partial accounting data options. Therefore, CO_2_ emissions associated with farm operations, N fertilization, and other chemical inputs should be taken into account in addition to those from GHG emissions and soil C sequestration while calculating net GWP and GHGI from agroecosystems.

**Fig 2:**
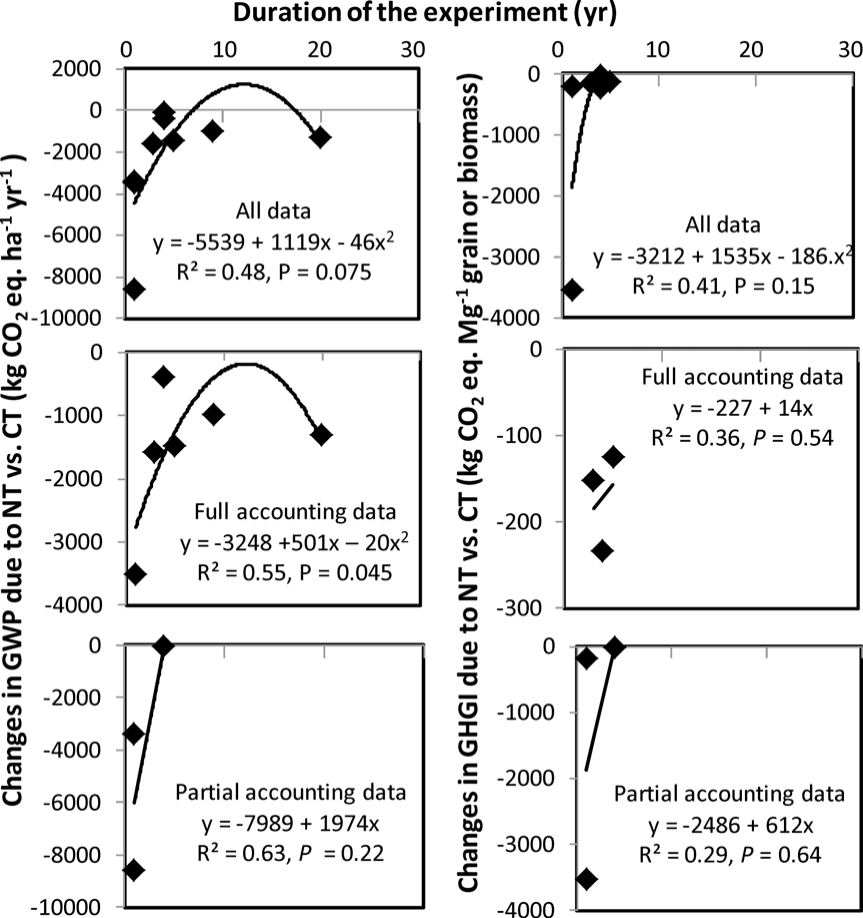
Changes in net global warming potential (GWP) and greenhouse gas intensity (GHGI) due to no-till (NT) vs. conventional till (CT) with the duration of the experiment using the soil organic C method. Full accounting data denote calculations of GWP and GHGI by accounting all sources and sinks of CO_2_ (N_2_O and CH_4_ emissions, farm operations, inputs, and soil C sequestration) and Partial accounting data, partial accounting of sources and sinks (N_2_O and CH_4_ emissions and/or soil C sequestration). All data denotes inclusions of full and partial accounting data.

Changes in GWP and GHGI due to no-till vs. conventional till with experiment duration were linear to curvilinear for all and full accounting data, but linear for partial accounting data (Fig 2). Availability of limited data resulted in fewer data points for GWP and GHGI in the partial accounting data. Both GWP and GHGI increased from 0 to 12 yr of experiment duration and then declined for all and full accounting data, but increased with increased duration of experiment for partial accounting data. This could be explained by several factors: (1) no-till can some time increases N_2_O emissions due to increased soil water content and denitrification compared with conventional till, especially in the humid region, thereby increasing GWP and GHGI [13, 16, 44], (2) the potential for soil C sequestration using no-till decreases and reaches a steady state as the duration of the experiment increases [44, 46], and (3) there is a high uncertainty in spatial and temporal variability in GHG emissions within and among regions due to variations in soil and climatic conditions and management practices [8, 16, 29, 30]. As C sequestration rate decreases due to increased C saturation with increased duration of the experiment [40, 44], GWP and GHGI may increase. When soil and climatic conditions, such as soil texture, annual precipitation, and average air temperature of the experimental sites were included in the multiple linear regressions, the relationships were dramatically improved (as indicated by higher R^2^ and lower *P* values) (Tables 7 and 8). While air temperature had a negative effect on GWP and GHGI, the effect of soil texture varied. As increased air temperature can increase GHG emissions due to accelerated mineralization of SOC, it is likely that increased temperature enhanced rate of SOC mineralization more than the rate of GHG emissions. As a result, temperature had a negative effect on GWP and GHGI. The potentials for reducing GWP and GHGI using no-till compared with conventional till, however, exist after 12 year. This is similar to that found by Six et al. [43] who reported that the benefit of no-till in reducing GWP and GHGI compared with conventional till was achieved only after 10 yr. Nevertheless, more long-term experiments are needed to relate the effect of tillage with duration of experiment on GWP and GHGI.

**Table 7:** Multiple linear regression analysis of net global warming potential (GWP) with management practices, duration of the experiment, total annual precipitation, mean annual air temperature, and soil texture from various locations.

Management practice	Intercept	Cropping intensity	N fertilization rate	Duration of the experiment	Total annual precipitation	Mean annual air temperature	Soil texture	R^2^	*P*
Tillage (NT vs. CT)†									
All data	-680	-----	-----	302	-2	-339	3185	0.89	0.031
Full accounting data	-359	-----	-----	278	-2	-94	-336	0.97	0.012
Partial accounting data	----------------------Model not full rank----------------------								
Cropping system‡									
Cropping intensity	2619	-2924	------	343	13	-1143	2751	0.86	0.003
Crop rotation vs. monocrop	281	------	------	-119	-2	144	-96	0.79	0.019
Corn-soybean vs. corn	1604	------	------	-113	-2	------	------	0.90	0.005
Small grain-legume vs. small grain	----------------------Model not full rank----------------------								
Perennial vs. annual crop	5127	------	------	-4824	-1	328	-757	0.99	0.002
N fertilization rate (kg N ha^-1^)†									
All data	-599	------	7.6	-188	0.06	175	528	0.75	0.011
Full accounting data	-2872	------	3.2	94	106	-107	-292	0.93	0.0001
Partial accounting data	8043	------	7.2	-3513	-5.7	932	-2765	0.84	0.0001
Combined management practice§									
Improved vs. traditional (SOC method)	-7695	-----	-----	757	7	-68	381	0.85	0.043
Improved vs. traditional (Soil respiration method)	-4753	-----	-----	59	4	-52	-1122	0.82	0.049

† Tillage is CT, conventional tillage; and NT, no-tillage. Full accounting data denotes calculation of GWP and GHGI by accounting all sources and sinks of CO_2_ (N_2_O and CH_4_ emissions, farm operations, inputs, and soil C sequestration). Partial accounting data denotes partial accounting of sources and sinks (N_2_O and CH_4_ emissions and/or soil C sequestration). All data denotes inclusions of full and partial accounting data.

‡ Small grains include wheat and barley. Cropping intensity was calculated based on number of crops grown in a year.

§ Combined management practices include combinations of tillage, cropping system, and N fertilization. Improved and traditional management were treatments with lowest and highest GWP and GHGI that were calculated by the soil organic C (SOC) or soil respiration method.

**Table 8:** Multiple linear regression analysis of net greenhouse gas intensity (GHGI) with management practices, duration of the experiment, total annual precipitation, mean annual air temperature, and soil texture from various locations.

Management practice	Intercept	Cropping intensity	N fertilization rate	Duration of the experiment	Total annual precipitation	Mean annual air temperature	Soil texture	R^2^	*P*
Tillage (NT vs. CT)†									
All data	1259	------	------	-80	2	-17	-1147	0.80	0.045
Full accounting data	1638	------	------	32	-44	5	------	0.94	0.015
Partial accounting data	9428	------	------	-126	-6	------	------	0.74	0.075
Cropping system‡									
Cropping intensity	2385	-1015	------	37	1	-328	757	0.94	0.0002
Crop rotation vs. monocrop	----------------------Model not full rank----------------------								
Corn-soybean vs. corn	----------------------Model not full rank----------------------								
Small grain-legume vs. small grain	----------------------Model not full rank----------------------								
Perennial vs. annual	----------------------Model not full rank----------------------								
N fertilization rate (kg N ha^-1^)†									
All data	713	------	-0.18	-94	0.0004	7.4	-91	0.77	0.063
Full accounting data	33.0	------	-0.23	40	0.81	-57	146	0.82	0.002
Partial accounting data	1034	------	0.33	373	-0.60	104	-336	0.73	0.0002
Combined management practice§									
Improved vs. traditional (Regular method)	-1335	------	------	27	15	625	-377	0.76	0.079
Improved vs. traditional(Alternative method)	----------------------Model not full rank----------------------								

† Tillage is CT, conventional tillage; and NT, no-tillage. Full accounting data denotes calculation of GWP and GHGI by accounting all sources and sinks of CO_2_ (N_2_O and CH_4_ emissions, farm operations, inputs, and soil C sequestration). Partial accounting data denotes partial accounting of sources and sinks (N_2_O and CH_4_ emissions and/or soil C sequestration). All data denotes inclusions of full and partial accounting data.

‡ Small grains include wheat and barley. Cropping intensity was calculated based on number of crops grown in a year.

§ Combined management practices include combinations of tillage, cropping system, and N fertilization. Improved and traditional management were treatments with lowest and highest GWP and GHGI that were calculated by the soil organic C (SOC) or soil respiration method.

### Effect of cropping system

**Fig 3:**
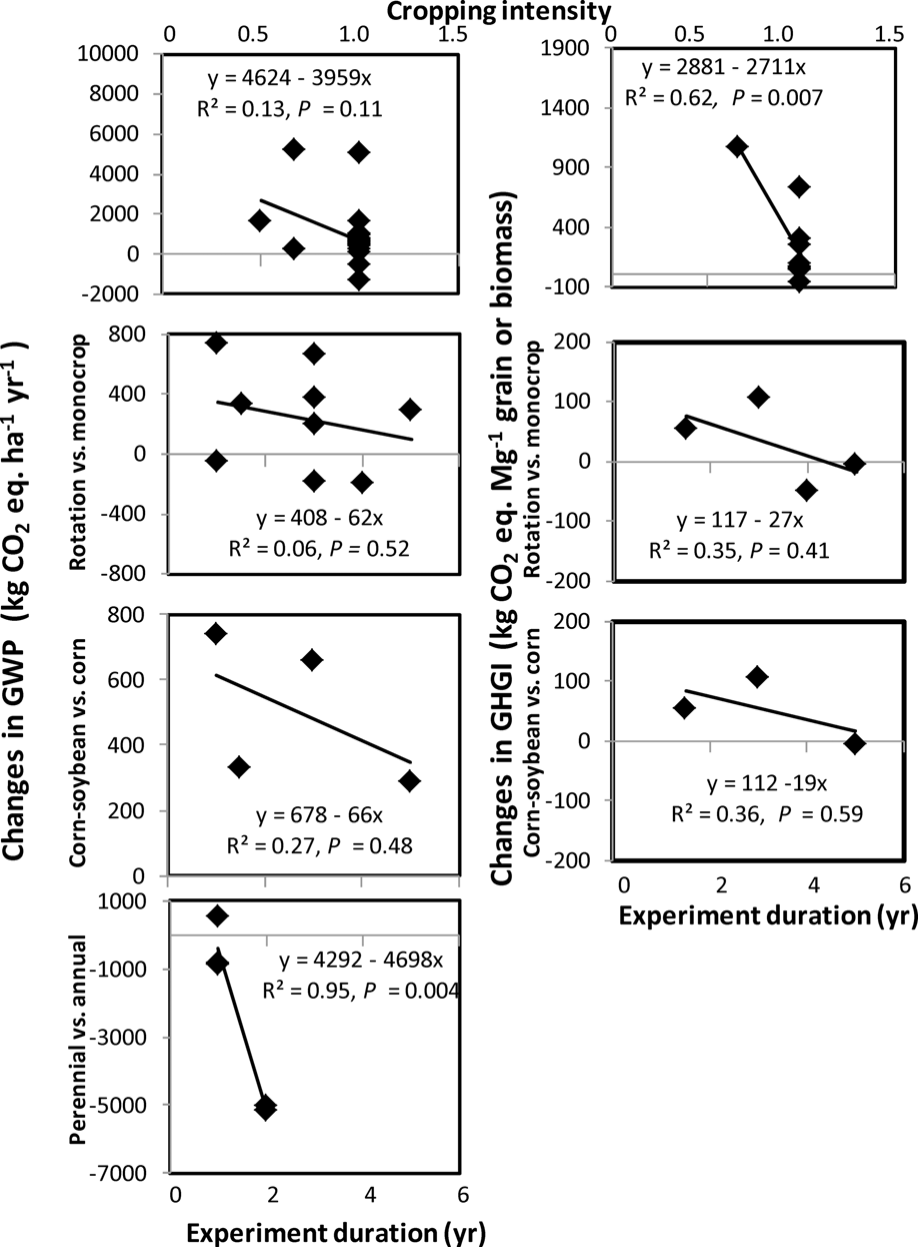
Changes in net global warming potential (GWP) and greenhouse gas intensity (GHGI) due to various cropping systems with the duration of the experiment using the soil organic C method. Because of the lack of sufficient data, only the all data option method of calculating GWP and GHGI were used for meta-analysis.

An evaluation of eleven experiments on cropping system containing small and large grain crops showed that crop rotation increased GWP by 46% and GHGI by 41% compared with monocropping (Tables 2 and 6). This was especially true for large grain crops, such as corn (*Zea mays* L.) and soybean (*Glycine max* L.), where GWP and GHGI were 215 and 325%, respectively, greater under corn-soybean than continuous corn (Table 6). In contrast, for small grain crops, such as barley (*Hordeum vulgare* L.) and pea (*Pisum sativum* L.), GWP was 22% lower under barley-pea than continuous barley. As cropping intensity increased, GWP and GHGI reduced (Table 6, Fig 3). Both GWP and GHGI were 168 and 215%, respectively, lower with perennial than annual cropping systems. Lack of sufficient data on crop yields prevented for comparison of some treatments for GHGI.

Increased ΔSOC due to greater crop residue returned to the soil reduced GWP and GHGI under continuous corn than corn-soybean rotation, although N fertilization rate to produce sustainable yield was higher in continuous corn [4, 8, 25, 26]. In contrast, greater N_2_O emissions following soybean increased GWP and GHGI in corn-soybean rotation [4, 8, 25, 26]. Under small grain crops, however, several researchers [29, 30, 43, 44, 47] have found that including legumes, such as pea and lentil (*Lens culinaris* L.), in rotation with nonlegumes, such as wheat (*Triticum aestivum* L.) and barley, reduced GWP and GHGI compared with continuous nonlegumes. They observed this because (1) no N fertilizer was applied to legumes compared with nonlegumes which required large amount of N fertilizers to sustain yields, as N fertilizer stimulates N_2_O emissions and (2) legumes supplied greater amount of N to succeeding crops due to higher N concentration when above- and belowground residues were returned to the soil and reduced N fertilization rate than nonlegumes. Sainju et al. [29, 30] also found that legume-nonlegume rotation increased ΔSOC because of increased turnover rate of plant C to soil C compared with continuous nonlegume. Greater number of experiments and magnitude of changes, however, resulted in higher GWP and GHGI with monocropping than crop rotation under large than small grain crops when values were averaged across experiments during data analysis.

Greater crop residue returned to the soil and increased ΔSOC reduced GWP and GHGI when cropping intensity was increased [28, 29]. Enhanced C sequestration with increased compared with reduced cropping intensity in the semiarid regions with limited precipitation has been well known [48, 49]. Several researchers [8, 28, 29] have found that fallowing or crop-fallow rotation increased GHG emissions and therefore GWP and GHGI compared with continuous cropping due to increased soil temperature and water content that enhanced microbial activity and absence of crops to utilize mineralized N during fallow. Perennial crops can reduce GWP and GHGI due to higher root biomass production [50, 51] and increased C sequestration [12, 45] compared with annual crops [8, 21, 28]. Perennial crops are not usually tilled or applied with fertilizers, herbicides, and pesticides, which reduce GHG emissions compared with annual crops [3].

Changes in GWP and GHGI due to crop rotation vs. monocrop, corn-soybean vs. continuous corn, and perennial vs. annual crop decreased with increased duration of experiment (Fig 3). This suggests that GWP and GHGI can be reduced in the long term by using improved cropping systems, such as crop rotation, intensive cropping, and perennial crops compared with monocropping, crop-fallow, and annual crops Although corn-soybean increased GWP and GHGI compared with continuous corn in the short term (Table 6), increased C sequestration rate in the long-term may reduce GWP and GHGI with corn-soybean with increased duration of the experiment. It may be possible that duration of obtaining C saturation may be shorter in continuous corn due to higher C sequestration rate than corn-soybean [40]. The relationships were further strengthened, especially for GWP, when soil and climatic conditions were accounted in the multiple linear regressions of GWP and GHGI with the duration of the experiment (Tables 7 and 8). Soil texture had a positive effect on GWP and GHGI for cropping intensity, but negative effect on GWP for crop rotation vs. monocrop and perennial vs. annual crop. The trend was opposite for mean air temperature while annual precipitation had small effect. Because the magnitude of ΔSOC is lower and time for C saturation is longer for cropping system than for tillage [44, 46], reduced GWP and GHGI for increased cropping intensity, crop rotation vs. monocrop, and perennial vs. annual crop with increased duration of experiment was probably due to increased C sequestration. The results showed that coarse texture soil can enhance GWP and GHGI compared with fine texture when cropping intensity was increased, but reduce GWP when crop rotation instead of monocropping or perennial instead of annual crop were used. The reverse was true in regions with higher than lower air temperature.

### Effect of nitrogen fertilization

**Fig 4:**
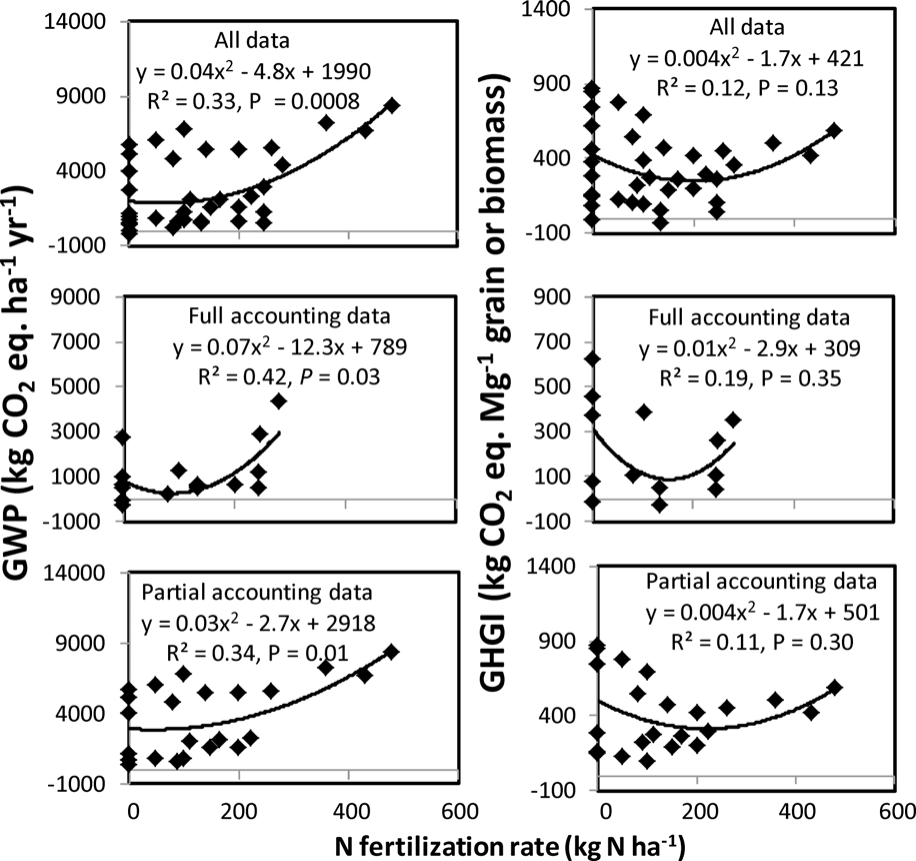
Relationship between N fertilization rate and net global warming potential (GWP) and greenhouse gas intensity (GHGI) using the soil organic C method. Full accounting data denote calculations of GWP and GHGI by accounting all sources and sinks of CO_2_ (N_2_O and CH_4_ emissions, farm operations, inputs, and soil C sequestration) and Partial accounting data, partial accounting of sources and sinks (N_2_O and CH_4_ emissions and/or soil C sequestration). All data denotes inclusions of full and partial accounting data.

The GWP decreased from 0 to ≤ 88 kg N ha^-1^ and then increased with increased N fertilization rate for full and partial accounting data as well as all data option (Table 3, Fig 4). Similarly, GHGI decreased from 0 to ≤ 213 kg N ha^-1^ and then increased with increased N rate for full and partial accounting and all data options. At lowest GWP and GHGI, N rates for the full accounting data were 88 and 145 kg N ha^-1^ compared with 45 and 213 kg N ha^-1^, respectively, for the partial accounting data.

Regardless of the data option used for calculating GWP and GHGI, results showed that increasing N rates to a certain level actually decreased GWP and GHGI, a case similar to that reported by various researchers [4, 18, 29, 30, 52, 53]. These N rates probably corresponded to crop N demand when crops used most of the soil available N, leaving little residual N in the soil that reduced N_2_O emissions and therefore GWP and GHGI. When N rates further increased, GWP and GHGI also increased, suggesting that excessive application of N fertilizers can induce net GHG emissions. Therefore, GWP and GHGI can be reduced if N fertilization rate can be decreased without affecting crop yields. Several researchers [3, 4, 29, 30] have reported that N rates to crops can be decreased to reduce GWP and GHGI without influencing crop yields. One practice is to use legume-nonlegume crop rotation where legume can reduce N rate to succeeding nonlegume by supplying more N compared with continuous nonlegume. Sainju et al. [29] have reported that N rate to dryland malt barley can be reduced by half by adopting malt barley-pea rotation compared while continuous malt barley while maintaining malt barley yield and quality. At 100 kg N ha^-1^ rate, GWP and GHGI were lower with the full accounting data (259 kg CO_2_ eq. ha^-1^ yr^-1^ and 119 kg CO_2_ eq. Ma^-1^ grain or biomass, respectively) than the partial accounting data (2948 kg CO_2_ eq. ha^-1^ yr^-1^ and 371 kg CO_2_ eq. Ma^-1^ grain or biomass, respectively). This is in contrast to the effect of tillage where GWP and GHGI were higher with full than partial accounting data. This suggests that, as with tillage comparison, GWP and GHGI values calculated by using the partial accounting data were overestimated and that CO_2_ equivalents associated with farm operations, N fertilization, and other chemical inputs should be accounted in addition to those from GHG emissions and ΔSOC when calculating net GWP and GHGI [21, 23, 24].

The relationships between GWP, GHGI, and N rate were further improved when duration of the experiment and soil and climatic factors were taken into account in the multiple linear regression (Tables 7 and 8). Duration of experiment and annual precipitation had positive effects, but air temperature and soil texture had negative effects on GWP when the full accounting data was used. With the partial accounting data, only air temperature had positive effect on GWP, but other factors had negative effects. For GHGI, the factors having negative effects were air temperature using the full accounting data and soil texture using the partial accounting data. Annual precipitation had minor effect on GWP and GHGI, a case similar to that observed for the effect of tillage.

### Effect of combined management practice

Using the all data option, the improved combined management practice that included no-till, diversified cropping system (crop rotation, increased cropping intensity, and perennial crop), and reduced N rate decreased GWP and GHGI by 70 to 88% compared with the traditional combined practice that included conventional till, less diversified cropping system (monocropping, crop-fallow, and annual crop), and recommended N rate (Tables 4 and 6). When compared with individual management practices, these reduction values were greater, such as 66 to 71% reductions for GWP and GHGI obtained with no-till vs. conventional till or -46 to -41% reductions with crop rotation vs. monocrop. Using the soil respiration method, reductions in GWP and GHGI were even higher, representing 133 to 158% reductions with the improved combined management practice compared with the traditional combined management practice (Tables 5 and 6).

These results clearly showed that the improved management practice can reduce GWP and GHGI compared with the traditional management practice, regardless of the methods used for calculating GWP and GHGI. Further reduction in the magnitudes of GWP and GHGI showed that the combined management practices may be more effective in reducing net GHG emissions than the individual practices, a case similar to those reported by various researchers [4, 8, 26, 29, 30]. The results also suggest that the soil respiration method may show greater GHG sink values for comparison of management practices than the SOC method [4, 28, 29, 30].

Using the SOC method, changes in GWP due to improved vs. traditional combined management practice increased from 0 to 3.5 yr of experiment duration and then decreased (Fig 4). Changes in GHGI, however, increased with increased duration of the experiment. In contrast, changes in GWP and GHGI using the soil respiration method were either not affected by or declined with the duration of the experiment. The relationships were further improved by including soil and climatic factors in the multiple linear regressions (Tables 7 and 8). As with the effect of individual management practices, some of the possible reasons for increased GWP and GHGI for improved vs. traditional combined management with increased duration of the experiment using the SOC method are: (1) high spatial and temporal variations of GHG emissions due to differences in soil and climatic conditions and management practices, (2) reduced potential for soil C sequestration with increasing duration of the experiment, (3) use of full or partial accounting data option for calculating GWP and GHGI, and (4) uncertainty in the methods of measuring GHG emissions, such as variations in type and size of static chambers, placement of chamber in the plot (row vs. inter-row or including vs. excluding plants in the chamber), time of GHG measurement during the day, and calculation of GHG fluxes (linear or nonlinear emissions with time). Results, however, indicate that GWP and GHGI can be reduced in the long term as duration of the experiment is increased, regardless of the method used. As a result, more long-term experiments may be needed to properly evaluate the effect of combined management practices on GWP and GHGI.

### Comparison of the methods of measurement

**Fig 5:**
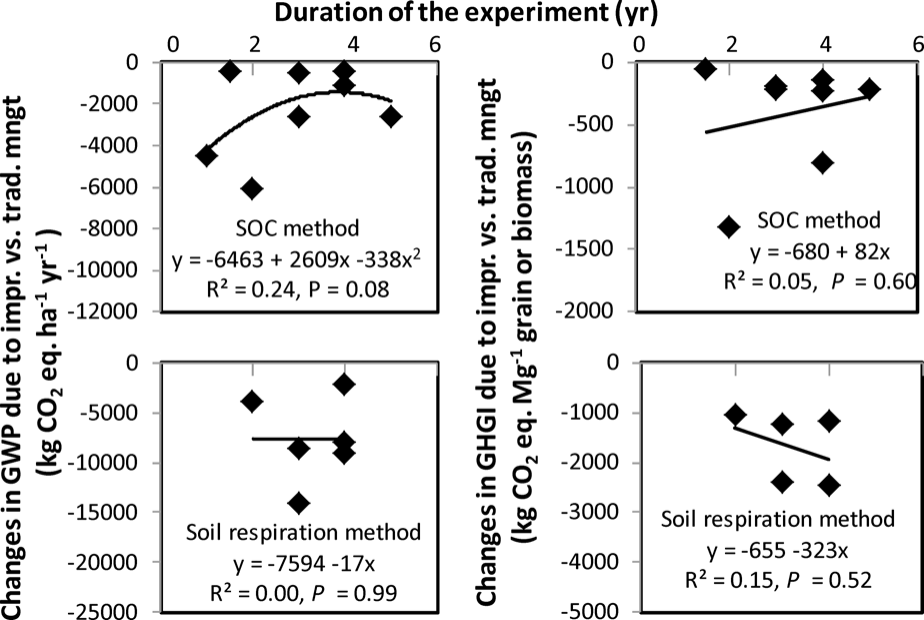
Changes in net global warming potential (GWP) and greenhouse gas intensity (GHGI) due to improved vs. traditional management practice with the duration of the experiment using the soil organic C and soil respiration methods. Because of the lack of sufficient data, only the all data option method of calculating GWP and GHGI were used for meta-analysis.

Both GWP and GHGI using SOC and soil respiration methods were lower with the improved combined management practice than the traditional combined management practice (Table 6). The GWP and GHGI measured with the soil respiration method were three to four times lower than those with the SOC method. Furthermore, changes in GWP and GHGI due to combined improved vs. combined traditional management practice were either not affected or decreased with the duration of experiment with the soil respiration method, but increased with the SOC method (Fig 5). This indicates that improved management practices may act more towards GHG sink than the traditional management practices using the soil respiration method compared with the SOC method. Similar results have been reported by various researchers [4, 28, 29, 30] who observed that most treatments were sources (or positive values) of GWP and GHGI when measured by using the SOC method, but sinks (or negative values) when measured by the soil respiration method. It is difficult to examine with the limited amount of data at present about which method provides efficient and accurate measurement of GWP and GHGI because of limitations, such as high variability in GHG emissions, slow changes in soil organic C levels, and differences in crop yields from year to year due to climatic conditions. More studies, however, are needed to accurately evaluate the effectiveness of each method on GWP and GHGI as affected by management practices.

Several researchers [4, 8, 29, 30] have reported that GWP and GHGI calculated by the soil respiration method had more variability than those calculated by the SOC method due to differences in CO_2_ emissions and crop residue production from year to year. This was against the results obtained in this study where coefficient of variations were 22% for GWP and 19% for GHGI calculated by the soil respiration method compared with 31 and 38%, respectively, calculated by the SOC method for combined management practices (Table 6). The SOC method is also subjected to high spatial variability due to interference from soil inorganic C, besides variability in GHG emissions [4, 8, 29, 30].

There are several benefits and drawbacks of the each method. The soil respiration method may provide quick results for GWP and GHGI compared with the SOC method, since only two years of experimentation are required when the amount of crop residue returned to the soil in the previous year is known. Information on C contributions from roots and rhozideposit from previous crop can further reduce GWP and GHGI calculated by this method [29, 30]. Such factors, however, are not usually measured in most experiments. Loss of previous crop residue due to the actions of wind and water or crop failure due to drought, especially in dryland cropping systems, can contribute significant errors in the calculation of GWP and GHGI in this method. Other needed information is soil respiration where the value of root respiration is excluded. Although the SOC method is a standard method and requires fewer parameters for calculating GWP and GHGI, it may take long time to measure these parameters using this method, because the process of C sequestration is slow and ΔSOC depends on soil and climatic conditions. In some cases, the benefits of management practices, such as no-till compared with conventional till, in reducing GWP and GHGI by the SOC method may not be realized after 10 yr [44]. While soil respiration and the amount of crop residue returned to the soil were the driving factors for GWP and GHGI in the soil respiration method, N_2_O emissions were the dominant factor in the SOC method. This study showed that GWP and GHGI calculated by the soil respiration method have potentials to decrease with increased duration of the experiment, which are in contrast to those obtained by the SOC method (Fig 5).

The notion that the soil respiration method showed greater reductions in GWP and GHGI than the SOC method may sometime provide false conclusions, especially during dry years when crop yields can be lower and GHG emissions can be higher, resulting in net CO_2_ source [29, 30]. During years with above-average precipitation, crop yields and the amount of crop residue returned to the soil can be greater, resulting in lower GWP and GHGI as measured by this method. In contrast, C sequestration is largely controlled by soil and climatic conditions among regions in the SOC method, although C input from crop residue can influence ΔSOC. Carbon sequestration rate can be higher in fine than in coarse-textured soil [29, 30]. Similarly ΔSOC can be greater in cold than in warm regions or higher in irrigated than dryland cropping systems. These factors add uncertainty in the measurements of GWP and GHGI in the SOC method. Both methods, however, showed that improved management practices, such as no-till continuous cropping with optimum N fertilization rate, can reduce GWP and GHGI compared with traditional practices, such as conventional till with crop-fallow and recommended N fertilization rate, a case similar to that reported by various researchers [4, 29, 30].

## Conclusions

Analysis of available global data revealed that improved management practices, such as no-till, diversified cropping systems, and reduced N fertilization rate, either as individually or in combination, reduced GWP and GHGI compared with traditional management practices, such as conventional till, less diversified cropping system, and recommended N rate. Changes in GWP and GHGI due to tillage practices were greater than changes due to cropping systems and N rates. Improved combined management practices further reduced GWP and GHGI compared with improved individual management practices. Adopting improved management practices for a longer period can further reduce GWP and GHGI. The GWP and GHGI values can be overestimated when indirect GHG emissions due to farm operations, N fertilization, and other chemical inputs were not accounted for. Both soil respiration and SOC methods showed similar results of measuring GWP and GHGI as affected by management practices. Although the soil respiration method may provide quick results for GWP and GHGI which can be higher for improved management practices than measured by the SOC method, greater variability in GHG measurements and crop yields from year to year suggest that more long-term studies are needed to accurately measure the effect of management practices on GWP and GHGI using both methods.

## Abbreviations

GHG: greenhouse gas
GHGI: greenhouse gas intensity
GWP: global warming potential
SOC: soil organic C
ΔSOC: soil C sequestration rate
