# Expression of Concern: A Global Meta-Analysis on the Impact of Management Practices on Net Global Warming Potential and Greenhouse Gas Intensity from Cropland Soils

**DOI:** 10.1371/journal.pone.0268102

**Published:** 2022-05-02

**Authors:** The PLOS ONE Editors

The PLOS Data Availability Policy requires that, with rare prior exception, all data underlying the findings described in an article are fully available without restriction.

The Data Availability Statement for this article [1] states that all relevant data are within the paper. However, the minimal data set underlying the findings of the study were not published with the article or deposited in a public repository at the time of publication. During editorial follow up on this issue, the corresponding author stated that the underlying data are no longer available.

The *PLOS ONE* Editors issue this Expression of Concern to alert readers that the data are unavailable and hence this article does not comply with *PLOS ONE*’s Data Availability Policy.
